# Malnutrition elevates delirium and ICU stay among critically ill older adult COVID-19 patients

**DOI:** 10.3389/fmed.2024.1259320

**Published:** 2024-05-10

**Authors:** Zahra Gholi, Masoud Rezaei, Zahra Vahdat Shariatpanahi, Reza Momen, Mehdi Fallah Bagher Shaidaei, Mostafa Gholami, Simin Aghaee, Hakimeh Eskandari Sabzi, Mohammad Reza Rajabi

**Affiliations:** ^1^Minimally Invasive Surgery Research Center; Iran University of Medical Sciences, Tehran, Iran; ^2^Nursing and Midwifery Care Research Center, School of Nursing and Midwifery, Iran University of Medical Sciences, Tehran, Iran; ^3^Cardiovascular Nursing Research Center, Rajaie Cardiovascular Medical and Research Center, Tehran, Iran; ^4^Department of Clinical Nutrition and Dietetics, National Nutrition and Food Technology Research Institute, Faculty of Nutrition and Food Technology, Shahid Beheshti University of Medical Sciences, Tehran, Iran; ^5^MSc in Critical Care nursing, Instructor, Critical Care Nursing Department, Faculty of Nursing, Aja University of Medical Sciences, Tehran, Iran; ^6^Clinical Research Development Unite, Ganjavian Hospital, Dezful University of Medical Sciences, Dezful, Iran; ^7^Nursing and Midwifery Care Research Center, Health Management Research Institute, Iran University of Medical Science, Tehran, Iran; ^8^Department of Pediatric Nursing, School of Nursing, Student Research Committee, Yasuj University of Medical Sciences, Yasuj, Iran; ^9^Dept. of Pediatric Nursing, School of Nursing and Midwifery, Ahvaz Jundishapour University Medical Sciences, Ahvaz, Iran, Ahvaz, Iran; ^10^Department of Cardiology, School of Medicine, Shahed University, Tehran, Iran

**Keywords:** malnutrition, COVID-19, older adult, critically ill patients, delirium

## Abstract

**Background and aim:**

Malnutrition among intensive care unit (ICU) patients is associated with a higher risk of mortality and prolonged hospitalization. However, the influence of malnutrition on severe outcomes of ICU patients with coronavirus disease 2019 (COVID-19) is unclear. By evaluating the effect of malnutrition on the outcomes of COVID-19 in the ICU in older adult patients, this study will contribute to new knowledge of assessing the nutritional status and recovery of these patients.

**Methods:**

In the current single center prospective study, 310 critically ill COVID-19 patients with an age range of ≥65 years were recruited. Data on demographic characteristics, laboratory parameters, comorbidities, medications, and types of mechanical ventilation were collected in the first 24 h of ICU admission. Malnutrition was defined based on the two-step approach of the Global Leadership Initiative on Malnutrition (GLIM) scale at baseline. During the 45 days after the baseline, we collected data on delirium incidence, mortality, invasive mechanical ventilation (IMV) requirement, length of ICU and hospital admission, and re-hospitalization.

**Results:**

In this study, the prevalence of malnutrition was 63.4% among critically ill COVID-19 patients. During the 45-day follow-up, 190 (61.3%) COVID-19 deaths were recorded among the baseline 310 patients. After controlling for potential confounders, malnutrition was associated with an increased risk of delirium so malnourished COVID-19 patients had a significantly 35% higher risk of delirium than those without malnutrition (HR: 1.35, 95% CI: 1.01–1.83). Such a significant association was not for COVID-19 mortality and IMV requirement. In addition, malnutrition was associated with a significantly 84% greater odds of prolonged ICU admission (OR: 1.84, 95% CI: 1.09–3.10). No significant association was seen between malnutrition and re-hospitalization and also prolonged hospital admission.

**Conclusion:**

Malnutrition was associated with an increased risk of delirium and prolonged ICU admission among critically ill older adult COVID-19 patients. Prevention, diagnosis, and treatment of malnutrition could be a key component in improving outcomes in these patients.

## Introduction

As a serious life-threatening issue, humankind has encountered Coronavirus Disease 19 (COVID-19) pandemic, caused by SARS-CoV-2-virus (1). According to the online WHO COVID-19 dashboard, 305 million people have been infected until January 2022, of which about 5.5 million patients eventually died (2). Although this disease may have severe outcomes among all age groups, it has the highest severity among older adult patients (3). Older adult COVID-19 patients have a higher risk of mortality, prolonged hospitalization, and the requirement for mechanical ventilation compared with younger patients. It is necessary to detect potential factors contributing to severe outcomes among older adult COVID-19 patients.

Previous studies have shown that comorbidities (cardiovascular disease, hypertension, diabetes, obesity, and chronic obstructive pulmonary disease), organ failure, and obesity have a role in severe outcomes of COVID-19 patients (3–6). Also, malnutrition may be involved. In addition to poor dietary intake (7), malnutrition might be caused by numerous conditions including inflammation, anorexia, and prolonged admission to the intensive care unit (ICU) (6, 8–13). It is estimated that 67% of ICU patients with COVID-19 have one of the malnutrition criteria. It is well known that malnutrition is associated with a higher risk of mortality and prolonged hospitalization. However, these associations among ICU patients with COVID-19 are not clear. Some studies indicated that malnourished COVID-19 patients had a higher risk of mortality and prolonged hospitalization compared with patients without malnutrition (14, 15), while other studies revealed a non-significant association (16).

The associations of malnutrition with other outcomes of ICU COVID-19 patients such as delirium and IMV requirement might be important. Delirium is a condition that affects the brain and delirious patients have some cognitive symptoms such as trouble focusing (called inattention), sudden changes in behavior, and confusion. Delirium is prevalent among 50% of ICU patients. Malnutrition could affect the brain’s nutrition, which is worth noting as a possible underlying mechanism of delirium. Only one study assessed the link between malnutrition and delirium among COVID-19 patients and reported a non-significant association (17). Also, data on the associations of malnutrition with IMV requirement and re-hospitalization are lacking. Therefore, the current study was done to assess the association between malnutrition and clinical outcomes of COVID-19 disease among older adult ICU patients.

## Materials and methods

### Study design and participants

This single-center prospective study was conducted in one of the hospitals in Tehran, Iran. At the time of the prevalence of COVID-19, this center was considered by the government as a general referral treatment center for patients with COVID-19. This study was conducted between August, 2021 and January, 2022 on critically ill older adult patients ≥65 years old with COVID-19 admitted to the ICU. The COVID-19 infection was diagnosed by chest CT scan lesions and positive reverse transcriptase polymerase chain reaction (RT-PCR) test.

Based on the manual classification of the disease of COVID-19 (6th edition) published by the National Health Commission of China (18), critically ill patients with COVID-19 were defined based on having the following criteria: (1) respiratory failure requiring mechanical ventilation; (2) septic shock; (3) Having at least one organ failure that requires care and treatment in the ICU. Men and women ≥65 years of age and willingness to participate in the study were the inclusion criteria. Patients who: (1) have been admitted to the ICU for the second time due to COVID-19; (2) had a history of neurological disorders, mental illnesses, cognitive disorders, dementia or delirium and cognition disorders; (3) had end-stage liver disease (ESLD) and end-stage renal disease (ESRD), advanced cancer or the presence of any brain damage and other severe comorbidities were not included in the study.

In order to avoid bias in data collection and to check the effectiveness of prescribed treatments in the intensive care unit, patients whose length of stay in the ICU was less than 48 h or who died during this period were excluded from the study. 392 older adult patients with COVID-19 were included in the study. At baseline, we gathered information on patients’ demographics, laboratory results, nutritional status, blood pressure, comorbidities, medications, and kinds of mechanical ventilation (the first day of ICU admission). Patients were monitored both during and 45 days after their first baseline admission to the ICU. During the follow-up period, data were collected on delirium incidence, mortality, IMV requirement, re-hospitalization, and duration of ICU or hospital admission for each patient.

### Ethics statement

Each participant signed an informed consent form in writing. The patient’s first-degree relatives had to sign the document if the patient wasn’t conscious. The use of medical record data for the current investigation was assured to patients in compliance with privacy rules. The Shahid Beheshti University of Medical Sciences Ethics Committee in Tehran, Iran gave its approval to the project. The ethical guidelines outlined in the 1964 Declaration of Helsinki and its later amendments were used as the basis for our work.

### Sample size calculation

The sample size was obtained using PASS 15.0 software, which is designed to determine the sample size and power of the studies. Determining the sample size according to the hazard ratio (HR) equal to 1.2 and with a probability of 95% (*α* = 0.05) and a power of 80% and a 60% incidence of 60-day mortality in critically ill patients with COVID-19 hospitalized in ICU, 272 patients were calculated. It was determined by considering the drop-out of patients during the study, 392 patients were enrolled for the sample size.

### Baseline assessment

During the first 24 h of admission to the ICU, demographic characteristics, laboratory parameters, nutritional status, vital signs (respiration rate, blood pressure, heart rate), comorbidities, drugs prescribed to control infection, and the need for mechanical ventilation as influencing variables were collected.

### Demographic and clinical characteristics

Information related to demographic characteristics and population variables included age, gender, marital status and education, anthropometry (height, weight and body mass index), history of smoking, history of concomitant diseases, vital signs (i.e., respiration rate, heart rate, blood pressure systolic and diastolic), and the history of alcohol consumption was collected by evaluating the medical records of the electronic file in the hospital or by questionnaire, as well as by interviewing the patient or the patient’s companion. Body mass index (BMI) was determined as weight in kilograms divided by height in meters squared. In addition, by examining the medical records, about the accompanying diseases such as lung diseases (i.e., asthma, chronic obstructive pulmonary disease, etc.), hyperlipidemia, information on diabetes mellitus, high blood pressure, heart diseases, acute and chronic renal failure, liver disease, ear/eye disorders, incidence of organ failure from the time of entering in ICU was recorded.

The protocols for controlling COVID-19 disease and its symptoms were also documented. These protocols included medications and different kinds of mechanical ventilation [IMV, non-invasive mechanical ventilation (NIMV), high-flow nasal cannula, face mask]. APACHE II score is a well-known indicator of disease severity in adults hospitalized in ICU and has a close relationship with predicting mortality in critically ill patients. The range of this score is from zero to 71. acute physiology and chronic health examination II (APACHE II) scoring for each person based on age, type of background disease (chronic health condition) and 12 physiological variables including temperature, mean arterial blood pressure, number of breaths per minute, heart rate per minute, arterial blood pH, oxygenation level of the patient based on FiO2, percentage of hematocrit, number of white blood cells, level of consciousness of the patient based on GCS and serum levels of sodium, potassium and creatinine were calculated.

### Laboratory parameters

Data on fasting blood sugar (FBS), serum levels of inflammatory biomarkers [C-reactive protein (CRP) and interleukin-6 (IL-6)], albumin, creatinine, urea, bilirubin, and 25-hydroxy vitamin D3 [25 (OH)D3] were obtained from patients medical records on the first day of ICU admission. Magnesium, phosphorous, calcium, sodium, and potassium were among the electrolytes whose serum levels were evaluated. We also gather information on hematological elements such platelet, white blood cells (neutrophil, lymphocyte) and hematocrit. Since greater Lactate dehydrogenase (LDH) levels are linked to a 6-fold increased risk of developing severe COVID-19 disease, baseline LDH levels were assessed in previous studies (19).

### Nutritional assessment

At baseline, malnutrition was defined based on the Global Leadership Initiative on Malnutrition (GLIM) criteria (20, 21). According to these criteria, diagnosis of malnutrition requires at least 1 phenotypic criterion and 1 etiologic criterion. Phenotypic criteria include (1) weight loss >5% within the past 6 months or > 10% beyond 6 months, (2) low BMI (<18.5 kg/m^2^ if patients were < 70 years and < 20 kg/m^2^ if patients were > 70 years), and (3) reduced muscle mass that was defined as calf circumference less than 34 cm in men and less than 33 in women. Etiologic criteria include (1) reduced food intake or assimilation that was defined by consuming 50% of energy requirement for >1 week or any reduction in energy intake for >2 weeks or chronic gastrointestinal disorders that adversely impact food assimilation or absorption.

During ICU hospitalization, dietary supplements and enteral feeding of food solutions, including commercial formulas and enteral powders, were recorded.

### Follow-up

During the ICU stay, incidence of delirium, readmission to the ICU, need for invasive mechanical ventilation, and incidence of mortality in the intensive care unit and 45-day mortality were also recorded. In the present study, delirium was assessed daily by an experienced ICU physician using the CAM-ICU (Confusion Assessment Method for the Intensive Care Unit) questionnaire (22, 23). Patients were monitored daily using the Glasgow Coma Scale to help assess acute onset or change in mental status abnormalities. With the help of this tool, the 4 main features of delirium are (1) acute change or fluctuating course of the patient’s mental state; (2) inattention, (3) impaired level of consciousness and (4) disorganized thinking are examined. If features 1 and 2 plus feature 3 or 4 were present, the diagnosis of delirium was given to the patient.

The length of hospital stay ≥14 days and the length of stay in ICU ≥7 days were considered as the average number of days that patients spent in hospital and ICU.

### Statistical analysis

Data were reported as mean ± SD for normal-distributed continuous variables, median (interquartile range) for non-normally distributed continuous variables, and percent for categorical variables. To assess the differences between patients with and without malnutrition in terms of normal and non-normally distributed continuous variables, we used the independent sample t-test and Mann–Whitney test, respectively. To determine the differences for categorical variables, the Chi-square test was used. We obtained the hazard ratios (HRs) and 95% confidence intervals (95% CIs) of mortality, delirium, and IMV requirement among malnourished patients, compared with patients without malnutrition, using multivariable Cox proportional hazards models. In the time-to-event analysis, follow-up time was considered as the day that outcome occurred or the day that patient was followed up. To assess the associations of malnutrition with prolonged hospitalization (≥7 days for ICU admission and ≥ 14 days for hospital admission) and re-hospitalization after discharge, we used multivariable binary logistic regression. We included potential confounders including age, gender, smoking, pre-existing pulmonary diseases, alcohol consumption, BMI, and blood levels of white blood cell (WBC), albumin, vitamin D, CRP levels, and vaccination in the adjusted models. *Statistical analysis was performed with SPSS* version 21 statistic *software* package. *p-*values less than 0.05 were considered statistically significant.

## Results

**Figure 1:**
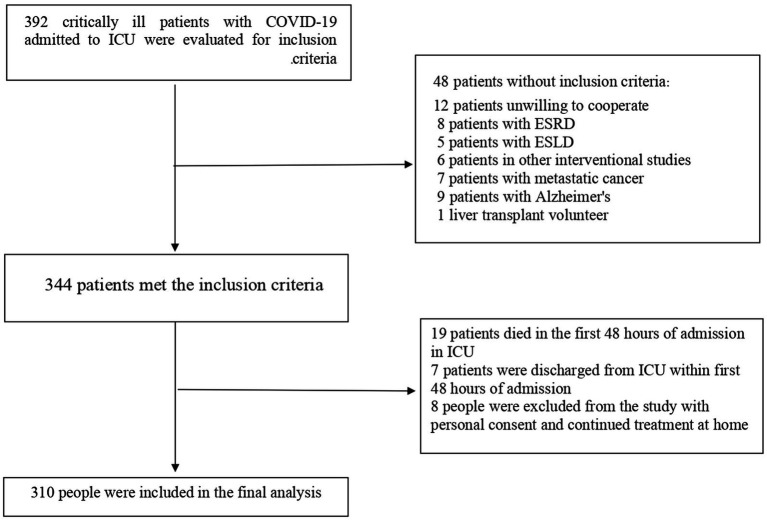
Study flow diagram of the 392 critically ill older adult COVID-19 patients included at baseline. 19 patients died during the first 48 h of hospitalization in ICU and therefore, were excluded. Moreover, 63 patients were excluded because they had missing data on exposure and outcome variables. Finally, 310 patients were included in the final analysis.

Of the 392 critically ill older adult COVID-19 patients included at baseline, 48 patients did not meet inclusion criteria. 19 patients died during the first 48 h of hospitalization in ICU, 7 patients were discharged from ICU within first 48 h of admission and therefore, were excluded. Moreover, 8 people were excluded from the study with personal consent and continued treatment at home. Finally, 310 patients with a mean age of 73.29 ± 6.91 years were included in the final analysis (Figure 1). We recorded 132 (42.6%) deaths during ICU admission and 190 (61.3%) deaths during 45 days after the baseline. Malnutrition was prevalent among 60.3% of patients. In addition, during the ICU admission, 217 (70.0%) cases of delirium were found and 53 (17.1%) patients required IMV therapy. Re-hospitalization occurred among 21% of patients during the 45 days of follow-up. All patients received antiviral and antibiotic drugs during ICU admission.

The baseline characteristics of COVID-19 patients with and without malnutrition are shown in Table 1. Patients with malnutrition had lower BMI, albumin levels, and higher APACHE II compared with well-nourished patients. Moreover, malnourished patients were more likely to be smokers, have organ failure, and need NIV and face masks in comparison to patients without malnutrition. No other significant differences were found in this regard.

**Table 1:** Characteristics of COVID-19 patients with and without malnutrition.

	Total (*N* = 310)	Without malnutrition (*n* = 123)	With malnutrition (*n* = 187)	*p*-value^*^
Demographic characteristics				
Age, y	73.29 ± 6.91	70.84 ± 6.21	74.90 ± 6.89	<0.001
Weight, kg	72.77 ± 10.50	78.12 ± 10.08	69.23 ± 9.21	<0.001
BMI, kg/m^2^	26.88 ± 3.28	28.69 ± 3.24	25.67 ± 2.71	<0.001
Female, %	41.3	48.0	36.9	0.05
Smokers, %	26.5	19.5	31.0	0.02
Married, %	78.6	86.2	73.7	0.009
University educated, %	11.7	16.3	8.6	0.04
Alcohol intake, %	7.7	5.7	9.1	0.27
Hematology				
WBC, 10^3^/μL	9.37 ± 4.51	8.93 ± 4.15	9.67 ± 4.72	0.15
Neutrophil, 10^3^/μL	83.43 ± 8.80	83.86 ± 9.01	83.15 ± 8.67	0.49
Lymphocyte, 10^3^/μL	12.20 ± 12.17	11.89 ± 13.01	12.42 ± 11.60	0.71
Platelet, 10^3^/μL	217.05 ± 71.11	221.52 ± 68.66	214.20 ± 72.66	0.38
Albumin, g/dL	3.05 ± 0.65	3.15 ± 0.66	2.98 ± 0.64	0.02
Hematocrit, %	35.78 ± 6.39	36.43 ± 6.23	35.36 ± 6.48	0.14
Biochemical assessment				
CRP, mg/L	87.41 ± 47.31	84.94 ± 51.17	89.03 ± 44.65	0.45
IL6, pg./mL	159.47 ± 216.41	165.76 ± 249.04	155.32 ± 192.57	0.67
Creatinine, mg/dL	1.40 ± 0.62	1.37 ± 0.69	1.42 ± 0.57	0.51
FBS, mg/dL	168.89 ± 53.83	167.39 ± 51.12	169.89 ± 55.66	0.68
Lactate dehydrogenase, U/L	518.70 ± 264.35	505.70 ± 288.18	527.24 ± 247.86	0.48
Vitamin D, ng/mL	30.03 ± 8.76	31.02 ± 9.10	29.37 ± 8.50	0.10
Bilirubin, mg/dL	0.83 ± 1.14	0.74 ± 0.88	0.90 ± 1.29	0.24
Urea, mg/dL	27.69 ± 16.15	27.03 ± 17.02	28.12 ± 15.58	0.56
Magnesium,	1.98 ± 0.39	2.02 ± 0.40	1.96 ± 0.37	0.18
Phosphorous,	3.02 ± 0.51	3.01 ± 0.55	3.02 ± 0.48	0.89
Calcium,	8.11 ± 0.57	8.14 ± 0.57	8.09 ± 0.57	0.44
Sodium,	136.43 ± 8.85	136.63 ± 3.76	136.29 ± 10.99	0.74
Potassium,	4.00 ± 0.69	4.00 ± 0.61	4.01 ± 0.74	0.96
Blood pressure				
SBP, mmHg	139.09 ± 22.20	135.74 ± 19.90	141.29 ± 23.38	0.03
DBP, mmHg	81.28 ± 15.30	80.11 ± 14.46	82.05 ± 15.82	0.27
Mean arterial pressure, mmHg	100.55 ± 16.69	98.65 ± 15.38	101.80 ± 17.42	0.10
Comorbidities				
Pulmonary disease, %	24.8	19.5	28.3	0.08
Hyperlipidemia, %	40.0	36.6	42.2	0.32
Diabetes, %	45.5	46.3	44.9	0.80
Hypertension, %	54.8	50.4	57.8	0.20
CVD, %	44.8	39.0	48.7	0.10
Hypothyroidism, %	20.8	17.4	23.1	0.23
Chronic renal disease, %	35.8	30.9	39.0	0.14
Liver disease, %	9.0	8.1	9.6	0.65
Stroke, %	5.5	2.5	7.5	0.06
Rheumatoid arthritis, %	1.3	1.7	1.1	0.66
Organ failure, %^a^	48.1	38.2	54.5	0.005
Ear problems, %	8.7	3.3	12.3	0.006
Eye problems, %	5.5	4.1	6.4	0.37
Medication				
Propofol, %	6.5	4.1	8.0	0.16
Opioid drugs, %	58.7	50.4	64.2	0.01
Glucocorticoids, %	65.5	72.4	61.0	0.04
Benzodiazepine, %	70.6	61.8	76.5	0.005
Vasopressor, %	46.8	56.9	40.1	0.004
Oxygen therapy at baseline				
Invasive MV, %	6.5	4.1	8.0	0.16
NIV, %	59.7	49.6	66.3	0.003
High flow nasal cannula, %	2.3	1.6	2.7	0.54
Face mask, %	59.0	72.4	50.3	<0.001
Outcomes during follow-up				
IMV therapy, %	17.1	10.6	21.4	0.013
Delirium, %	70.0	58.5	77.5	<0.001
Death during ICU admission, %	42.6	31.7	49.7	0.002
Death during 45 days, %	61.3	48.0	70.1	<0.001
Re-hospitalization, %	21.0	17.9	23.0	0.28
Acute renal failure, %	26.5	23.6	28.3	0.35
Hospitalization				
Length of hospital stay (day)	14 (10–19)	15 (10–19)	13 (10–19)	0.72
Length of ICU stay (day)	8 (6–10)	7 (5–9)	8 (6–10)	0.06
APACHE score	17 (11–21)	14 (10–19)	18 (13–21)	<0.001
Vaccination	49.7	48.3	52.7	0.79

Data are presented as mean ± SD for normal-distributed continuous variables, median (interquartile range) for non-normally distributed continuous variables, and percent for categorical variables. BMI, body mass index; WBC, white blood cell; IL-6, interleukin – 6; CRP, C-reactive protein; FBS, fasting blood sugar; SBP, systolic blood pressure; DBP, diastolic blood pressure; CVD, cardiovascular disease; MV, mechanical ventilation; NIV, non-invasive ventilation; SD, standard deviation. Obtained from independent sample *t*-test (normal-distributed continuous variables), Mann–Whitney test (non-normally distributed continuous variables), or chi-square test (categorical variables).

a Considered as the incidence of failure of ≥ 2 organs.

Multivariable-adjusted HRs (95% CIs) of delirium, COVID-19 mortality, and IMV requirement in relation to malnutrition are shown in Table 2. We found significant positive associations between malnutrition and risk of delirium (HR: 1.45, 95% CI: 1.09–1.92) and IMV requirement (HR: 1.94, 95% CI: 1.03–3.63). After considering potential confounders, observed association for IMV requirement became non-significant, however, delirium remained significant so that COVID-19 patients with malnutrition had a 35% higher risk of delirium compared with well-nourished patients (HR: 1.35, 95% CI: 1.01–1.83). Regarding COVID-19 mortality, we found no significant association either before or after controlling for potential confounders.

**Table 2:** Hazard ratios for some outcomes of COVID-19 patients with malnutrition compared to those without malnutrition.

	Without malnutrition	With malnutrition
Delirium		
Cases	72	145
Unadjusted	1.00	1.45 (1.09–1.92)
Model 1	1.00	1.38 (1.03–1.86)
Model 2	1.00	1.35 (1.01–1.83)
IMV therapy^a^		
Cases	13	40
Unadjusted	1.00	1.94 (1.03–3.63)
Model 1	1.00	1.29 (0.66–2.53)
Model 2	1.00	1.28 (0.65–2.51)
Death during 45 days		
Cases	59	131
Unadjusted	1.00	1.78 (0.83–3.81)
Model 1	1.00	1.22 (0.54–2.75)
Model 2	1.00	1.34 (0.58–3.12)
Death during ICU admission		
Cases	39	93
Unadjusted	1.00	1.36 (0.93–1.98)
Model 1	1.00	1.10 (0.74–1.64)
Model 2	1.00	1.04 (0.69–1.57)

Data are presented as HR (95% CI). IMV, invasive mechanical ventilation; WBC, white blood cell; CRP, C-reactive protein; HR, hazard ratio. Model 1: Adjusted for age, gender, smoking, and history of pulmonary diseases. Model 2: Further adjustments for WBC, vitamin D, CRP levels, vaccination. HRs were obtained from the Cox regression analysis.

a With considering IMV therapy at baseline.

Multivariate-adjusted odds ratios (ORs) and 95% CIs for prolonged hospitalization and re-hospitalization in relation to malnutrition are presented in Table 3. We found a significant positive association between malnutrition and prolonged hospitalization in ICU (OR: 1.72, 95% CI: 1.08–2.76). After controlling for potential confounders, malnourished patients had 84% higher odds of prolonged admission to ICU (>7 days) compared with those without malnutrition (OR: 1.84, 95% CI: 1.09–3.10). In terms of re-hospitalization and length of stay in the hospital, we found no significant association with malnutrition.

**Table 3:** Odds ratios for hospital outcomes of COVID-19 patients with malnutrition compared to those without malnutrition.

	Without malnutrition	With malnutrition
Hospital stay≥14 days		
Cases	68	93
Unadjusted	1.00	0.80 (0.51–1.26)
Model 1	1.00	1.09 (0.66–1.79)
Model 2	1.00	1.14 (0.69–1.90)
ICU stay≥7 days		
Cases	67	126
Unadjusted	1.00	1.72 (1.08–2.76)
Model 1	1.00	1.90 (1.14–3.16)
Model 2	1.00	1.84 (1.09–3.10)
Re-hospitalization		
Cases	22	43
Unadjusted	1.00	1.37 (0.77–2.43)
Model 1	1.00	1.31 (0.72–2.43)
Model 2	1.00	1.32 (0.71–2.46)

Data are presented as OR (95% CI). ICU, intensive care unit; WBC, white blood cell; CRP, C-reactive protein; OR, odds ratio. Model 1: Adjusted for age, gender, smoking, and history of pulmonary diseases. Model 2: Further adjustments for WBC, vitamin D, CRP levels, vaccination. ORs were obtained from the binary logistic regression.

## Discussion

In the current study, malnutrition was associated with an increased risk of delirium and prolonged ICU stay among critically ill COVID-19 patients. These associations were obtained after controlling for potential confounders. Regarding other COVID-19 outcomes including mortality, IMV requirement, and re-hospitalization, there was no significant association with malnutrition even after taking potential confounders into account.

Malnutrition is a highly prevalent disorder among hospitalized patients, particularly in ICU patients, and is associated with severe outcomes such as mortality. In the current study, 60.3% of patients were affected by malnutrition. Previous studies also reported a high prevalence of malnutrition among hospitalized COVID-19 patients. Bedock et al. reported a prevalence of 42.1% among hospitalized patients and 66.7% among ICU patients (24). The prevalence of malnutrition may be higher among older adult patients compared with younger patients. Therefore, the management of malnutrition among older adult COVID-19 patients is of great importance. The effect of low protein intake in the older adult, the importance of sulfur amino acids in their diet, low glutathione levels and parenteral nutrition low in cysteine, low gastric/digestive acidity, etc. may be related to malnutrition in the older adult and COVID-19 (25, 26).

In the current study, malnutrition was associated with an increased risk of delirium among COVID-19 patients. Delirium is referred to a neurocognitive syndrome representing different manifestations, including acute brain dysfunction with fluctuations in the basal mental state, inattention, disorganized thinking, or altered levels of consciousness (27). In contrast with our findings, Rebora et al. reported a prevalence of 14.3% for delirium among COVID-19 patients in a multi-center study and showed a non-significant association between malnutrition and delirium (17). The disparity might be explained by the different study populations in terms of gender, age group, and comorbidities. In addition, different criteria for the definition of malnutrition are another potential reason for the inconsistency. Also, different adjustments in the statistical analysis may be involved. Further studies are required to assess the link between malnutrition and delirium among ICU COVID-19 patients. Many factors can affect the occurrence of delirium. Considering that proper nutrition is necessary for the functioning of all organs of the body, the brain is an organ with high metabolic activity and nutritional needs, so the lack of nutrients in malnutrition affects the development of brain diseases and delirium (28, 29). Studies have shown that the lack of macronutrients, micronutrients and vitamins due to the disruption of neurotransmitters has an effective role in the occurrence of delirium and cognitive disorders (25, 26, 30). To date, only a few studies have shown the association of delirium with malnutrition (31, 32).

Serum albumin level is also known as an indicator of nutritional status and inflammation in predicting adverse outcomes of patients (33). The mechanism of the effect of serum albumin on reducing cognitive disorders has been attributed to the combination with beta-amyloid. This combination prevents the fibrosis of proteins by inhibiting the accumulation of beta-amyloid, and finally, it is associated with the prevention of cognitive disorders and Alzheimer’s (34).

Another outstanding result of the present study was the non-significant association between malnutrition and IMV requirement among COVID-19 patients. However, previous studies investigating the association among ICU patients presented different findings. In a study on patients with COVID-19 pneumonia, Porto et al. reported that malnourished patients were more likely to need IMV therapy compared with well-nourished patients (35). In the current study, the rate of IMV therapy among malnourished patients (8%) was two times more than well-nourished patients (4.1%). In addition, the positive association between malnutrition and risk of IMV requirement was significant in the unadjusted model. However, it became non-significant when taking potential confounders into account. The lack of significant association might be explained by the low number of IMV therapy among the study population. It has been demonstrated that malnutrition may adversely affect thoraco-pulmonary functions through ventilatory drive alteration, decreasing the ventilatory response to hypoxia, decreased mass, force, contractility, and endurace of the diaphragm, reducing respiratory muscle strength, diminishing the synthesis of alveolar surfactant, changing the humoral and cellular immunity, and increasing bacterial adhesion in the lower respiratory tract (36). It seems that further studies are needed to finalize the link between malnutrition and IMV requirement among COVID-19 patients.

We found no significant association between malnutrition and COVID-19 mortality. In line with our results, Sanchez-Rodriguez et al. reported a lack of significant association between malnutrition (using the geriatric nutritional risk index) and COVID-19 mortality among older adult patients (16). However, in some studies, a significant positive association was reported in this regard (37). The observed inconsistency might be due to different scales used for the malnutrition definition. Moreover, different sample sizes and different adjustments are other reasons for the observed different results. On the other hand, the clinical manifestations of malnutrition and accompanying diseases are different in each patient and can have a different effect on the mortality rate.

According to our findings, malnutrition had a significant positive association with prolonged ICU stay (≥7 days). In a review article, Powers et al. concluded that a 20% increase in the prevalence of malnutrition in ICU is associated with prolonged ICU stay (38). In addition, Nigatu et al. reported that malnutrition was highly associated with prolonged length of hospital stay (39). However, contrary to our study, Osooli et al. reported that the duration of ICU stay was not affected by nutritional status among ICU patients with different conditions (40). As a well-known health issue, hospital malnutrition has been characterized for more than 20 years ago (41). Different study populations, recruiting patients with different conditions, and defining malnutrition by different scales are possible reasons for the observed disparity among the previous studies. In the current study, we found no significant association between malnutrition and prolonged hospital stay, unlike ICU stay. It might be explained by limited hospital admission capacities in Iran during the COVID-19 epidemic. Because of the high incidence of COVID-19 and limited hospital beds, patients may be discharged as soon as possible. Therefore, the lack of significant association between malnutrition and prolonged hospital stay should be considered with caution.

Some limitations must be taken into consideration when interpreting our results. Due to the lack of a gold standard for malnutrition diagnosis, the misclassification of patients in terms of nutritional status is unavoidable. It should be noted that the GLIM scale is the best among other scales used for malnutrition diagnosis. In addition, we could not estimate the amount of reduction in food intake and absorption as an item required to complete the GLIM questionnaire. This may increase misclassification bias. This may increase the misclassification bias. Despite controlling for several potential confounders, our results might be still affected by residual confounders such as lifestyle information, income level, place of residence, healthy food intake, exercise and therapeutic protocols used for controlling COVID-19. These factors can affect the severity of disease symptoms and recovery over a shorter period of time, and unfortunately, in this study, due to the conditions of the patients and their companions, it was not possible to collect these items.

Despite efforts to minimize measurement error through rigorous training of data collectors and regular calibration of measurement tools, concerns about measurement accuracy persist.

Vaccination may distort our findings on the link between malnutrition and clinical outcomes of COVID-19 patients. However, the rate of vaccination was not different across the patients with and without malnutrition. Since this was a single-center study and the sample size was low, the generalizability of our findings to all COVID-19 patients should be done with caution.

In conclusion, the present study highlighted the positive association of malnutrition with the risk of delirium and prolonged ICU stay among older adult COVID-19 patients. However, the associations of malnutrition with other clinical outcomes of COVID-19 patients such as IMV requirement and mortality were not significant and remained still questionable. Taken together, malnutrition should be given high attention in the treatment protocols of COVID-19 patients. Future studies should assess the rate of single-nutrient deficiencies among COVID-19 patients and their associations with clinical outcomes of this disease. Because nutritional deficiency consists of severely reduced levels of one or more nutrients, making the body unable to normally perform its functions and thus leading to an increased risk. Prevention, diagnosis, and treatment of malnutrition could be a key component in improving outcomes in these patients.

### What is already known on this subject?

Previous studies have shown that malnutrition have a role in severe outcomes of COVID-19 patients. It is well known that malnutrition is associated with a higher risk of mortality and prolonged hospitalization. However, these associations among ICU patients with COVID-19 are not clear. Few studies have been done on older adult patients. Only one study assessed the link between malnutrition and delirium among COVID-19 patients and reported a non-significant association. Also, data on the associations of malnutrition with IMV requirement and re-hospitalization are lacking. Therefore, the current study was done to assess the association between malnutrition and clinical outcomes of COVID-19 disease among older adult ICU patients.

### What this study adds?

We found that malnutrition was associated with an increased risk of delirium and prolonged ICU stay among critically ill COVID-19 patients. These associations were obtained after controlling for potential confounders. Regarding other COVID-19 outcomes including mortality, IMV requirement, and re-hospitalization, there was no significant association with malnutrition even after taking potential confounders into account.

## Data availability statement

The raw data supporting the conclusions of this article will be made available by the authors, without undue reservation.

## Ethics statement

The studies involving humans were approved by The Shahid Beheshti University of Medical Sciences Ethics Committee in Tehran, Iran. The studies were conducted in accordance with the local legislation and institutional requirements. Written informed consent for participation in this study was provided by the participants' legal guardians/next of kin.

## Author contributions

ZGh: Methodology, Writing – original draft, Writing – review & editing, Investigation. MR: Methodology, Investigation, Writing – original draft. ZVSh: Methodology, Writing – original draft. RM: Methodology, Writing – original draft. MF: Methodology, Writing – original draft. MG: Methodology, Writing – original draft. SA: Methodology, Writing – original draft. HE: Methodology, Writing – original draft. MRR: Investigation, Methodology, Writing – original draft, Writing – review & editing.
